# Expression Profiling of CYP1B1 in Oral Squamous Cell Carcinoma: Counterintuitive Downregulation in Tumors

**DOI:** 10.1371/journal.pone.0027914

**Published:** 2011-11-16

**Authors:** Shalmali Pradhan, M. N. Nagashri, K. S. Gopinath, Arun Kumar

**Affiliations:** 1 Department of Molecular Reproduction, Development and Genetics, Indian Institute of Science, Bangalore, Karnataka, India; 2 Department of Surgical Oncology, Bangalore Institute of Oncology, Bangalore, Karnataka, India; Virginia Commonwealth University, United States of America

## Abstract

Oral Squamous Cell Carcinoma (OSCC) has a very flagitious treatment regime. A prodrug approach is thought to aid in targeting chemotherapy. CYP1B1, a member of cytochrome P450 family, has been implicated in chemical carcinogenesis. There exists a general accordance that this protein is overexpressed in a variety of cancers, making it an ideal candidate for a prodrug therapy. The activation of the prodrug facilitated by CYP1B1 would enable the targeting of chemotherapy to tumor tissues in which CYP1B1 is specifically overexpressed as a result reducing the non-specific side effects that the current chemotherapy elicits. This study was aimed at validating the use of CYP1B1 as a target for the prodrug therapy in OSCC. The expression profile of CYP1B1 was analysed in a panel of 51 OSCC tumors, their corresponding normal tissues, an epithelial dysplasia lesion and its matched normal tissue by qRT-PCR, Western blotting and Immunohistochemistry. *CYP1B1* was found to be downregulated in 77.78% (28/36) tumor tissues in comparison to their corresponding normal tissues as well as in the epithelial dysplasia lesion compared to its matched normal tissue at the transcriptional level, and in 92.86% (26/28) of tumor tissues at the protein level. This report therefore clearly demonstrates the downregulation of *CYP1B1* at the transcriptional and translational levels in tumor tissues in comparison to their corresponding normal tissues. These observations indicate that caution should be observed as this therapy may not be applicable universally to all cancers and also suggest the possibility of a prophylactic therapy for oral cancer.

## Introduction

Oral squamous cell carcinoma (OSCC) is the most common head and neck cancer, with a worldwide incidence of 640,000 new cases annually [1]. Disappointingly, survival rates of OSCC have not improved since the last many decades. Both smoked and smokeless forms of tobacco are major inducers of OSCC [2]. Cigarette smoke has been shown to upregulate cytochrome P450 (CYP1B1 and CYP1A1) under *in vitro* conditions as well as in smokers [3]–[5]. The *CYP1B1* gene is transcriptionally activated by polycyclic aromatic hydrocarbons which are major constituents of cigarette smoke and tobacco, making it responsive to smoked and smokeless (chewing) tobacco [6]–[8]. CYP1B1 plays a role in the bioactivation of chemically diverse tobacco related procarcinogens to reactive metabolites. Thus, the expression of CYP1B1 is considered to be an important determinant of carcinogenesis [9]. It has been observed that CYP1B1 is overexpressed in a wide array of human tumors compared to their respective normal tissues [8], [10], [11]. Allelic variations in *CYP1B1* have also been shown to modulate the incidence of several types of cancers [12], [13]. CYP1B1 has also been implicated in aiding resistance to tamoxifen, docetaxel and flutamide [11]. Thus, CYP1B1 appears to play an important role incarcinogenesis as well as therapeutics.

Differential expression of CYP1B1 in tumor cells creates an opportunity to develop potential strategies that may involve either inhibiting CYP1B1 activity (which as stated above activates procarcinogens) or using the metabolic activity of CYP1B1 to activate essentially non-toxic prodrugs (e.g., resveratrol and aryl oxime) to cytotoxic compounds. Therapies based on these prodrugs are in preclinical evaluations [14] and clinical trials for colorectal cancer, melanoma and follicular lymphoma (http://clinicaltrials.gov/ct2/results?term=resveratrol). The overall aim of this study was to validate CYP1B1 as a therapeutic target in OSCC. Although previous studies have suggested CYP1B1 protein expression to be tumor specific [9], [15], our observations clearly indicate that the protein is not only expressed in normal as well as tumor tissues, but is also downregulated in oral tumor tissues.

## Results

### Determination of CYP1B1 mRNA levels

**Figure 1:**
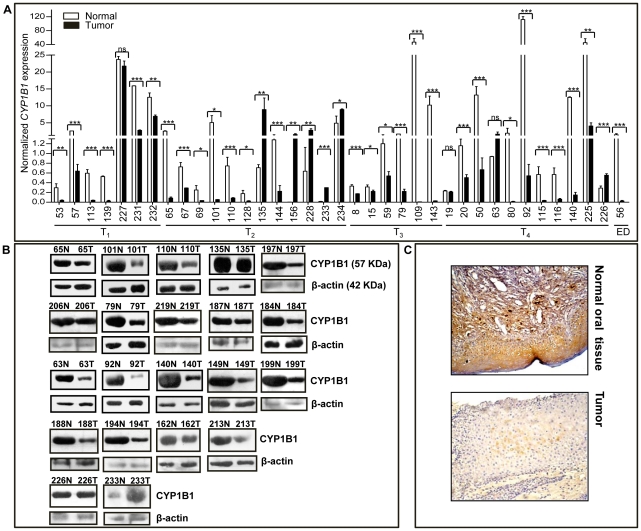
Expression profiling of CYP1B1 in OSCC. (**A**) qRT-PCR analysis of *CYP1B1* in 36 matched normal and tumor pairs, and one epithelial dysplasia sample with its corresponding normal tissue. Each bar represents an average of duplicate reactions. The numbers on the X-axis represent patient numbers. T_1_, T_2_, T_3_ and T_4_ represent the different grades of tumors according to the TNM (tumor, node and metastasis) classification. ED represents epithelial dysplasia. ***  = p<0.001, *  = p<0.05 and ns  =  not significant. (**B**) The Western blot analysis of 21 matched normal oral and tumor tissues. Note the downregulation of CYP1B1 in oral tumors compared to their corresponding normal oral tissues in all but two cases. The numbers represent the patient numbers. N and T represent normal and tumor tissues respectively. β-actin was used as a loading control. (**C**) Immunohistochemical staining for CYP1B1 in matched normal and tumor tissues from patient 136. Note the reduced expression of CYP1B1 in the tumor tissue as compared its corresponding normal tissue.

All the 36 matched tumor and normal tissue pairs analysed showed the expression of *CYP1B1* mRNA (Figure 1A). *CYP1B1* showed statistically significant downregulation across the tumor stages at the transcriptional level in 28/36 pairs as analyzed by qRT-PCR (Figure 1A). As seen from the Figure 1A, five tumor tissues (patients 135, 156, 228, 233 and 234) showed significant upregulation of *CYP1B1* mRNA, while the differential regulation of *CYP1B1* was not significant in the matched tumor and normal tissue samples of patients19, 63 and 227. Similar to a majority of the tumor tissues, the epithelial dysplasia (ED) lesion also showed significant downregulation of *CYP1B1*.

### Determination of CYP1B1 protein levels

The expression of CYP1B1 protein was analyzed by Western blotting in 21 pairs, of which 10 werealso used for qRT-PCR analysis. The protein expression levels as observed from the Western blots corroborate well with the expression profile of the *CYP1B1* mRNA levels as determined by using qRT-PCR in all but three cases (Figure 1B). In patients 63 and 135, though the mRNA levels were higher in tumor tissues than their corresponding normal tissues (Figure 1A), tumor tissues showed downregulation of CYP1B1 at the protein level (Figure 1B). In patient 226, *CYP1B1* was upregulated at the mRNA level in the tumor tissue, but equal expression of CYP1B1 protein was observed in normal and tumor tissues (Figure 1B). On the whole, CYP1B1 protein was downregulated in 19/21 tumor tissues in comparison to their corresponding normal tissues (Figure 1B). One sample (patient 226) did not show any difference in CYP1B1 level between normal and tumor tissues, while in patient 233, it was upregulated in tumor tissue as compared to the normal tissue (Figure 1B).

### Determination of CYP1B1 protein levels by immunohistochemistry

Immunohistochemistry was carried out for a total of 7 matched normal and tumor tissue sections, of which three cases (patients 139, 143 and 144) had also been used for qRT-PCR analysis. All the cases analysed showed lower staining intensity in tumor sections as compared to the matched normal sections (Figure 1C). It is evident from the Immunohistochemistry as well as the Western blot analysis that the CYP1B1 proteinis expressed in all normal tissues analysed and ispredominantly downregulated in tumor tissues.

## Discussion

This study has investigated the expression profile of *CYP1B1* in a panel of 51 OSCC cases and an epithelial dysplasia case. *CYP1B1* was found to be downregulated at the mRNA level as determined by qRT-PCR in 77.78% (28/36) of the tumor tissues in comparison to their corresponding normal tissues and an epithelial dysplasia case (Figure 1A). The differences in the CYP1B1 protein levels between the matched normal and tumor tissues affirm the differences seen at the transcriptional level for all except three cases: patient numbers 63, 135 and 226 (Figure 1A and 1B). The protein expression was analysed using two different techniques, namely Western blotting and Immunohistochemistry. On the whole, 92.86% (26/28) of the cases showed a clear-cut downregulation of CYP1B1 in tumor tissues as compared to their matched normal tissues (Figure 1B and 1C and Table S1). Further, the expression profile of CYP1B1 does not seem to be dependent on the gender of the patients since the downregulation of CYP1B1 is observed in both males as well as in females (Table S1). For example, the male patient 110 and the female patient 101 show downregulation of CYP1B1 at both the mRNA and the protein levels (see Figure 1B and Table S1). Thus unlike the previous reports in mouse Leydig cells and endometrial cancer [16], [17], the downregulation of CYP1B1 does not seem to be governed by estrogen. However, similar to our study, the absence of gender differences in the pattern of CYP1B1 expression has also been observed recently in OSCC [18], [19]. On the whole, differential expression of CYP1B1 was found to be independent of the site of the lesion, gender of the patient, TNM status and other etiological factors such as tobacco and alcohol consumption (Figure S1). The observation of CYP1B1's expression in all the normal tissues along with its downregulation in tumor tissues is contradictory to numerous earlier reports regarding the expression profile of CYP1B1 in other cancers of brain, kidney, lung, colon, bladder, breast, prostate, endometrium, ovaries and esophagus [8]–[11].

Nagraj et al. [3] observed that the basal transcript level of *CYP1B1* was highest in the non-malignant and pre-malignant oral cell lines compared to the oral cancer cell line. Also the rate and magnitude of induction of *CYP1B1* by cigarette smoke condensate was the highest in the non-malignant normal cells followed by the pre-malignant cells and then the tumor cells [3]. These observations, though made in an *in vitro* system, corroborate well in terms of the difference observed in the levels of *CYP1B1* transcription between normal and tumor tissues.

Our study is not the first one to document the expression profile of CYP1B1 in OSCC [18], [19]. However, contrasting observations have been made with respect to the expression of CYP1B1 in OSCC tumor tissues. For example, unlike our observations, Shatalova et al. [19] have documented the upregulation of CYP1B1 in a study on the head and neck squamous cell carcinoma which included only 19.5% of oral lesions. This difference might be due different kinds of oral lesions examined by us and Shatalova et al. [19]. On the other hand, similar to our observations, Kolokythas et al. [18] showed downregulation of *CYP1B1*at the mRNA level only in OSCC from oral brush cytology samples. Ours is the only study documenting the CYP1B1 downregulation in matched tumor and normal OSCC samples at both the transcriptional and the translational levels (Figure 1 and Table S1).

The downregulation of CYP1B1 has also been reported in melanoma and endometrial cancer [17], [20], although many other reports have contradictory observations and demonstrated the upregulation of CYP1B1 in endometrial tumors [11]. The reason behind the observed downregulation of *CYP1B1* in tumor tissues can only be speculated upon. *CYP1B1* is regulated by the aryl hydrocarbon receptor (AhR) and AhR nuclear translocator (ARNT) complex (AhR/ARNT), apart from many other key transcription factors [11]. The AhR/ARNT complex functions as an intracellular receptor for a variety of environmental toxicants like polycyclic aromatic hydrocarbons (e.g., benzo[a]pyrene), polyhalogenated hydrocarbons, dibenzofurans, and the potent small-molecular-weight toxicant, 2,3,7,8-tetrachlorodibenzo-p-dioxin (TCDD or dioxin) [21]. Most of these are tobacco derived carcinogens [21]. The binding of these ligands to the AhR/ARNT complex enables its translocation to the nucleus where it transcriptionally activates a plethora of genes, including *CYP1B1* [11]. The induction of *CYP1B1* in the oral mucosa has also been shown to be majorly due to tobacco associated polycyclic aromatic hydrocarbons (PAH) [4], [7] anda major regulator associated with PAH is AhR. As seen from Table S1, a majority of the patients involved in this study are tobacco consumers. The transcription induced by AhRis known to be repressed by AhRR (AhR repressor) [22]. Constitutive expression of AhRR has been shown to make cells non-responsive to agents that would otherwise induce *CYP1A1* [23]. Similar to *CYP1A1*, the induction of *CYP1B1* also is governed by AhR. Thus, the levels of AhRR can be speculated to govern downregulation of *CYP1B1* in tumor tissues. Apart fromAhRR, epigenetic modifications such as promoter methylation of *CYP1B1* in tumor tissues could also account for the downregulation of CYP1B1. Interestingly, this mechanism has been documented in melanoma [20]. Further, the level of miR-27b was shown to be inversely correlated with the expression of CYP1B1 in breast cancer tissue [24]. Thus, miRNAs such as miR-27b could also be contributing factors for its regulation. However, these possibilities need to be explored in future.

The tumor-specific expression pattern of CYP1B1 has been suggested to be exploited for the development of targeted anticancer therapies [9], [14]. Our study indicates that caution should be observed as this therapy may not be applicable universally to all cancers. The fact that CYP1B1 expression is higher in the normal tissues might aid to the development of chemopreventive drugs for oral cancer. In the light of the knowledge that CYP1B1 plays a role in carcinogenesis [9], [11], [12], drugs that inhibit CYP1B1 might be useful prophylactic agents. During a clinical examination of the oral cavity of a patient, it is often possible to distinguish normal tissue, premalignant lesion (e.g., leukoplakia) and malignant tumor. An Indian survey showed that 80% of the malignancies of the oral cavity arise from premalignant lesions, such as leukoplakia, erythroplakia, and oral submucous fibrosis [25]. The presence of such premalignant lesions would enable prophylactic treatments which otherwise are not possible in most of other cancers. Apart from drugs that inhibit CYP1B1, prodrugs that are activated by CYP1B1 might also be used as prophylactic drugs since they would result in depletion of the cells expressing it, enabling reduction in the risk to develop a malignant lesion.

In summary, the CYP1B1 downregulation in oral tumor tissues as compared to their matched normal tissues contradicts many studies which state that CYP1B1 is overexpressed in tumors [8], [10], [11]. This downregulation is consistent over a range of tumor stages (TNM) and independent of the gender of the patient, site of the lesion and etiology of the carcinogenesis. On the basis of our observations, we suggest that a level of caution should be observed for treatments based on CYP1B1 overexpression in tumors.

## Materials and Methods

### Ethics statement

This research followed the tenets of the Declaration of Helsinki, and the informed written consent for research was obtained from the patients enrolled in the study following the approval from the ethics committee of the Bangalore Institute of Oncology.

### Clinical material

A total of 51 matched oral tumor and normal tissues, one epithelial dysplasia (ED) along with its matched normal tissue as well as paraffin-sections from seven matched tumor and normal tissues (Table S1) were ascertained at the Bangalore Institute of Oncology, Bangalore, and R.L. Jalappa Hospital and Research Center, Kolar, both located in the state of Karnataka, India. The specimens from patients undergoing either surgical resection or biopsy of the oral cancerous lesion and the adjacent normal mucosa (approximately 5 cm from the lesion) were collected in RNAlater™ (Sigma-Aldrich, USA) immediately upon surgery. The patients were not under any treatment at the time of the surgery. Tumors were classified according to TNM (Tumor, Node and Metastasis) classification based on the UICC (Union for International Cancer Control, Switzerland; http://www.uicc.org/resources/how-use-tnm-classification).

### Total RNA isolation and first-strand cDNA synthesis

Total RNA was isolated from 36 matched tumor and normal tissue pairs, and one epithelial dysplasia lesion along with its matched normal using TRI Reagent® (Sigma-Aldrich, USA). The first-strand cDNA was synthesized using the RevertAid™ H Minus First Strand cDNA synthesis kit (MBI Fermentas, Canada) using 1.5 µg RNA and random hexamer primers in a 20 µl reaction as per the manufacturer's protocol.

### Quantitative RT-PCR analysis

Following primers for quantitative RT-PCR (qRT-PCR) analysis were synthesized by Sigma-Aldrich (Bangalore): *GAPDH* forward (5′- GAAGGTGAAGGTCGGAGTC-3′) and reverse (5′-GAAATCCCATCACCATCTTC-3′) [26]; and, *CYP1B1* forward (5′TACCGGCCACTATCACTGACATCTTC-3′) and reverse (5′TGGATCAGGTCGTGGGGAGGGACC-3′). Complementary DNA (cDNA) equivalent to 10 ng of total RNA was used for qRT-PCR reactions. All the qRT-PCR reactions were carried out using Dynamo SYBR green mix (Finnzymes, Finland) in an ABI Prism® 7000 sequence detection system (Applied Biosystems, USA). The analysis has been done using the SDS 2 software (Applied Biosystems, USA). *GAPDH* was used as a normalizing control [26]. Normalized values for *CYP1B1* expression in tumor and normal tissues were calculated as follows: ΔC_(t)_ Tumor  =  C_(t)CYP1B1_ - C_(t)GAPDH_, and ΔC_(t)_ Normal  =  C_(t)CYP1B1_ - C_(t)GAPDH_. Where, C_(t)_ is the cycle number at which the fluorescence intensity crosses the default threshold value of 0.2 and ΔC_(t)_ represents the *CYP1B1* expression normalized to *GAPDH*. ΔΔC_(t)_ (relative *CYP1B1* expression) is equal to ΔC_(t)_ Tumor - ΔC_(t)_ Normal.

### Western hybridization

Tissue lysates were prepared from matched normal and tumor tissues separately by homogenizing the tissue (∼100 mg) in CellLytic™ MT Mammalian Tissue Lysis/Extraction Reagent (Sigma-Aldrich, USA) supplemented with 1X protease inhibitor cocktail (Sigma-Aldrich, USA). Equal amount of protein was resolved on a 10% SDS-PAGE and then transferred onto a PVDF membrane (PerkinElmer Life Sciences, USA). For Western hybridization, rabbit polyclonal anti-CYP1B1 (Santa Cruz Biotechnology, USA) and mouse monoclonal β-actin (Sigma-Aldrich, USA) primary antibodies were used. The signals were detected using HRP-conjugated goat anti-rabbit or goat anti-mouse secondary antibodies (Bangalore Genei, India), Western Lightning Chemiluminescence Reagent Kit (PerkinElmer Life Sciences, USA) and X-ray films (Kodak, India).

### Immunohistochemistry

Formalin-fixed and paraffin-embedded tissue sections (5 µm thick) prepared on poly-L-lysine solution (Sigma-Aldrich, USA) coated microscopy slides were dewaxed in xylene and rehydrated to water through decreasing percentages of ethanol prepared in distilled water. The antigen retrieval step was carried out using sodium citrate buffer (2.96 g/l sodium citrate prepared in distilled water, pH = 6). Endogenous peroxidase activity was quenched with freshly prepared 1% H_2_O_2_ in 1X PBS (phosphate buffered saline/0.05 M Na_2_HPO_4_, 0.05 M NaH_2_PO_4_ and 0.15 M NaCl, pH 7.4) for 30 min at room temperature in dark. Nonspecific antibody binding was blocked with 2% BSA in 1X PBST (phosphate buffered saline with 0.1% tween) for 1 hr at room temperature in a humidified chamber. Rabbit polyclonal anti-CYP1B1 primary antibody was allowed to hybridize overnight at 4°C. Post washes, HRP-conjugated goat anti-rabbit secondary antibody was added. After washes, the sections were exposed to 1 mg/ml DAB (Sigma-Aldrich, USA) in 1X PBS and 1% H_2_O_2_. Subsequent to washes, the sections were counterstained with hematoxylin, progressively dehydrated using ethanol gradation and mounted in DPX mountant (S.D. Fine-Chem Ltd., India). The sections were analysedunder 40x using a Olympus microscope.

### Statistics

The significance of differences in the mRNA expression levels in the qRT-PCR analysis was assessed by two-tailed student's t-test using the GraphPad PRISM® version 5 software (GraphPad Software Inc., USA). For determining the statistical significance of the correlationbetween the relative expression of *CYP1B1* and gender of the patient, site of the lesion, and tobacco and alcohol consumption by the patient, we used the unpaired t-test with Welch's correction. While one-way ANOVA followed by a post-test (Tukey's multiple comparison test) was used to determine whether the levels of *CYP1B1* were dependent on the TNM of the tumors. A probability value of p<0.05 was assumed to be significant.

## Supporting Information

Figure S1 **Correlation of** ***CYP1B1*** **expression with clinicopathological features of the patients.** (**A**) The graph shows the relative expression of *CYP1B1*in OSCC tumor tissues compared to their matched normal tissues in female and male patients.Note that the relative expression of *CYP1B1* was observed to be independent of the gender of the patient. (**B**)The graph shows the relative expression of *CYP1B1* in various grades of the tumors as per the TNM classification. T_1_, T_2_, T_3_ and T_4_ represent the different grades of tumors according to the TNM (tumor, node and metastasis) classification. Note that the status of *CYP1B1* expression did not show any significant correlation with the TNM of the tumor tissues. (**C**)The graph shows the relative expression of *CYP1B1* with respect to the site of the lesions. Note that the expression of *CYP1B1* did not vary significantly between the BM (buccal mucosa) and other sites of the lesion. Since most of the lesions were from the buccal mucosa, the comparison was carried out between buccal mucosa and all the other sites combined. (**D**) The graph shows that the relative expression of *CYP1B1* did not significantly differ between consumers and non-consumers of tobacco, areca nut, betel nut and alcohol. Abbreviation: ns represents statistically non-significant data.
(TIF)

Table S1 **Detailedclinicopathological features of patients included in the study.** Information regarding the 51 cases and one epithelial dysplasia (ED) included in the study with respect to age, sex, tumor node metastasis (TNM) classification, site of lesions, etiology (habits) and, the expression profile of CYP1B1 at the mRNA level and the protein levels are given. Abbreviations: BM, buccal mucosa; N>T, downregulation in tumor tissues; N<T, upregulation in tumors; ns, statistically non significant; WB, Western blotting; and IHC, immunohistochemistry.
(PDF)
