# Supplementary figures and images for: Expression Profiling of CYP1B1 in Oral Squamous Cell Carcinoma: Counterintuitive Downregulation in Tumors
Source: PLoS One. 2011 Nov 16;6(11):e27914. doi: 10.1371/journal.pone.0027914 (PMC3218060; doi:10.1371/journal.pone.0027914)

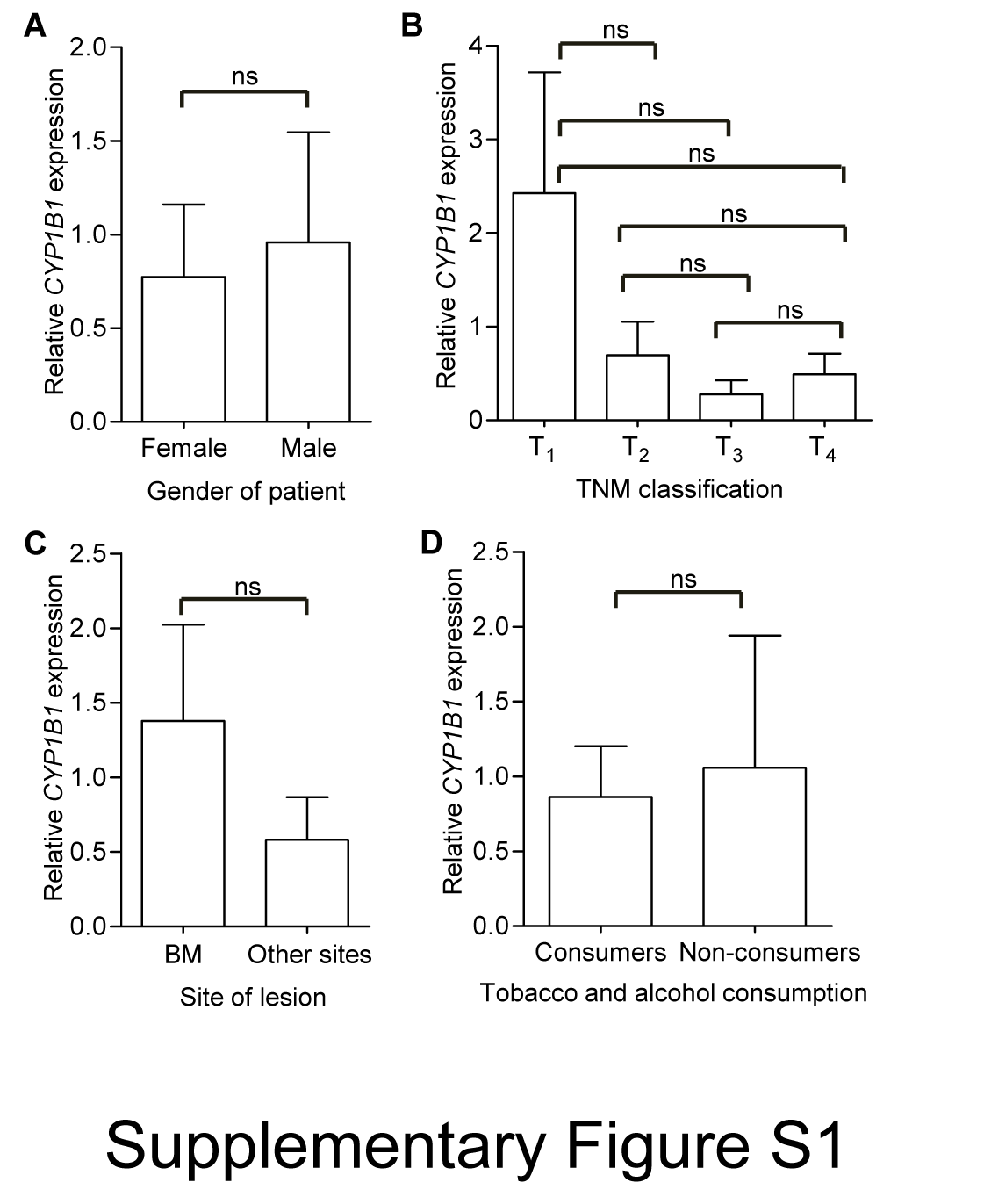

Supplement: Figure S1 — Correlation of CYP1B1 expression with clinicopathological features of the patients. (A) The graph shows the relative expression of CYP1B1in OSCC tumor tissues compared to their matched normal tissues in female and male patients.Note that the relative expression of CYP1B1 was observed to be independent of the gender of the patient. (B)The graph shows the relative expression of CYP1B1 in various grades of the tumors as per the TNM classification. T1, T2, T3 and T4 represent the different grades of tumors according to the TNM (tumor, node and metastasis) classification. Note that the status of CYP1B1 expression did not show any significant correlation with the TNM of the tumor tissues. (C)The graph shows the relative expression of CYP1B1 with respect to the site of the lesions. Note that the expression of CYP1B1 did not vary significantly between the BM (buccal mucosa) and other sites of the lesion. Since most of the lesions were from the buccal mucosa, the comparison was carried out between buccal mucosa and all the other sites combined. (D) The graph shows that the relative expression of CYP1B1 did not significantly differ between consumers and non-consumers of tobacco, areca nut, betel nut and alcohol. Abbreviation: ns represents statistically non-significant data. (TIF) [file pone.0027914.s001.tif]
