# Supplementary material for: Expression Profiling of CYP1B1 in Oral Squamous Cell Carcinoma: Counterintuitive Downregulation in Tumors
Source: PLoS One. 2011 Nov 16;6(11):e27914. doi: 10.1371/journal.pone.0027914 (PMC3218060; doi:10.1371/journal.pone.0027914)
Supplement: Table S1 — Detailedclinicopathological features of patients included in the study. Information regarding the 51 cases and one epithelial dysplasia (ED) included in the study with respect to age, sex, tumor node metastasis (TNM) classification, site of lesions, etiology (habits) and, the expression profile of CYP1B1 at the mRNA level and the protein levels are given. Abbreviations: BM, buccal mucosa; N>T, downregulation in tumor tissues; N<T, upregulation in tumors; ns, statistically non significant; WB, Western blotting; and IHC, immunohistochemistry. (PDF) [file pone.0027914.s002.pdf]

Supplementary Table S1

Sr. No	Patient no.	Age/ Sex	TNM	Site of lesion	Habits	*CYP1B1* expression (mRNA)	CYP1B1 expression (protein)
1	8	50F	$T_3N_1M_0$	Lower alveolus	Tobacco chewing	N>T	-
2	15	51F	$T_3N_1M_0$	BM	Tobacco, areca nut, betel nut chewing	N>T	-
3	19	48F	$T_4N_{2b}M_0$	BM	Betel quid chewing	ns	-
4	20	50F	$T_4N_1M_0$	BM	Betel nut chewing	N>T	-
5	50	50M	$T_4N_{2b}M_0$	BM	Tobacco chewing	N>T	-
6	53	38F	$T_1N_0M_0$	BM	Tobacco chewing	N>T	-
7	56	50F	ED	BM	Tobacco chewing	N>T	-
8	57	62F	$T_1N_0M_0$	BM	Tobacco chewing	N>T	-
9	59	70F	$T_3N_1M_0$	Retromolar trigone	Betel nut chewing	N>T	-
10	63	40F	$T_{4a}N_1M_x$	BM	Betel nut chewing	ns	N>T (WB)
11	65	55F	$T_2N_1M_x$	BM	Tobacco chewing	N>T	N>T (WB)
12	67	38F	$T_2N_1M_0$	BM	Tobacco chewing	N>T	-
13	69	70F	$T_2N_1M_0$	BM	Tobacco chewing	N>T	-
14	79	67F	$T_3N_1M_x$	BM	Tobacco chewing	N>T	N>T (WB)
15	80	67F	$T_4N_0M_0$	Floor of the mouth	Tobacco chewing	N>T	-
16	92	50F	$T_{4a}N_{2b}M_0$	BM	Tobacco chewing	N>T	N>T (WB)
17	101	48F	$T_2N_1M_x$	BM	Tobacco chewing	N>T	N>T (WB)
18	109	45M	$T_3N_1M_0$	BM	Tobacco chewing	N>T	-
19	110	45M	$T_2N_1M_0$	BM	Smoking and alcohol	N>T	N>T (WB)
20	113	80F	$T_1N_0M_0$	BM	Betel nut chewing	N>T	-
21	115	55F	$T_4N_1M_0$	BM	Tobacco chewing	N>T	-
22	116	40M	$T_4N_0M_0$	BM and retromolar trigone	Tobacco chewing	N>T	-
23	128	60F	$T_2N_0M_0$	BM	Tobacco chewing	N>T	-
24	135	41M	$T_2N_0M_0$	BM	Betel quid chewing	N<T	N>T (WB)
25	136	50F	$T_1N_0M_0$	Tongue	Nil	-	N>T (IHC)
26	139	71F	$T_1N_1M_0$	BM	Tobacco chewing	N>T	N>T (IHC)
27	140	70M	$T_4N_1M_0$	Lip	Tobacco chewing	N>T	N>T (WB)
28	142	62M	$T_3N_0M_0$	Floor of the mouth	Cigarette smoking	-	N>T (IHC)
29	143	43M	$T_3N_1M_0$	BM	Tobacco chewing	N>T	N>T (IHC)
30	144	40M	$T_2N_1M_0$	BM and angle of the mouth	Cigarette smoking	N>T	N>T (IHC)
31	149	50M	$T_4N_{2c}M_x$	BM	Tobacco chewing	-	N>T (WB)
32	156	75M	$T_2N_0M_0$	Tongue	Tobacco chewing	N<T	-
33	162	55F	$T_2N_0M_0$	BM	Tobacco chewing	-	N>T (WB)
34	178	50M	$T_1N_0M_0$	Tongue	Nil	-	N>T (IHC)
35	184	38M	$T_2N_1M_0$	Tongue	Betel nut chewing and alcohol	-	N>T (WB)
36	185	56M	$T_3N_0M_0$	BM	Nil	-	N>T (IHC)
37	187	83M	$T_3N_0M_0$	Maxilla	Cigarette smoking	-	N>T (WB)
38	188	33M	$T_3N_0M_0$	Palate	Nil	-	N>T (WB)
39	194	48F	$T_3N_0M_0$	Ginigvobuccal sulcus	Tobacco chewing	-	N>T (WB)
40	197	59M	$T_2N_0M_0$	BM	Cigarette smoking and alcohol	-	N>T (WB)
41	199	50M	$T_{4a}N_{2b}M_0$	BM	Tobacco chewing	-	N>T (WB)
42	206	44M	$T_3N_{2b}M_0$	Tongue	Tobacco chewing	-	N>T (WB)
43	213	37M	$T_3N_0M_0$	Tongue	Nil	-	N>T (WB)
44	219	68M	$T_3N_1M_0$	BM	Tobacco chewing	-	N>T (WB)
45	225	53F	$T_4N_0M_0$	BM	Betel quid chewing	N>T	-
46	226	55F	$T_4N_2M_0$	BM	Nil	N<T	N=T (WB)
47	227	28M	$T_1N_1M_0$	BM	Tobacco chewing and smoking	ns	-
48	228	54F	$T_2N_0M_0$	BM	Tobacco chewing	N<T	-
49	231	58M	$T_1N_1M_0$	Tongue	Nil	N>T	-
50	232	38M	$T_1N_0M_0$	Soft palate	Tobacco chewing and smoking	N>T	-
51	233	50F	$T_2N_0M_0$	BM	Tobacco chewing	N<T	N<T (WB)
52	234	58M	$T_2N_0M_0$	Mandible and tongue	Betel nut and betel quid chewing	N<T	-
